# Adherence to the OARSI recommendations for designing, conducting, and reporting of clinical trials in knee osteoarthritis: a targeted literature review

**DOI:** 10.1186/s12891-022-05116-z

**Published:** 2022-02-22

**Authors:** Charles D. Hummer, Yili Huang, Brendan Sheehan

**Affiliations:** 1 Premier Orthopaedics and Sports Medicine, 300 Evergreen Drive, Suite 200, Glen Mills, PA 19342 USA; 2 Zucker School of Medicine at Hofstra/Northwell, Northwell Phelps Hospital, Sleepy Hollow, NY USA; 3 Saint John Orthopaedics, Saint John, NB Canada

**Keywords:** Knee osteoarthritis, OARSI, Intra-articular therapies, Quality assessment, Randomized clinical trials

## Abstract

**Background:**

The Osteoarthritis Research Society International (OARSI) updated their guideline for clinical trials on knee osteoarthritis (KOA) in 2015, which contains recommendations for the conduct, design, and reporting of clinical trials. The purpose of this study was to assess the quality of clinical trials published between 2010 and 2020 investigating intra-articular interventions in patients with KOA using the OARSI recommendations.

**Methods:**

A targeted literature review was conducted to identify randomized controlled trials in patients with KOA receiving intra-articular interventions, published between 2010 and 2020. Included studies were assessed using the OARSI recommendations. For a comparison between the time periods before and after the introduction of the new OARSI recommendations, the year 2016 was selected as the cut-off.

**Results:**

One hundred forty-eight publications, representing 139 unique trials, were included in this review. Included studies adhered to between 9 and 24 recommendations (median: 19). The highest increase in adherence from studies published in 2016 or earlier compared to after 2016 was seen in the reporting and registration of trials and the use of structural outcome measures. Overall, adherence to the recommendations related to the collection of biochemical biomarkers and the use of structural outcome measures remained low.

**Conclusion:**

An improvement can be made in the conduct, design, and reporting of clinical trials for intra-articular therapies in KOA. Despite proper guidelines, quality of clinical trials varies, and the methodological deficiencies found are preventable and can be corrected. The quality of research should be considered when making treatment decisions for patients with KOA in clinical practice.

**Supplementary Information:**

The online version contains supplementary material available at 10.1186/s12891-022-05116-z.

## Background

As the most common disabling joint disease, osteoarthritis (OA) affects over 32.5 million Americans as of 2020 [1]. Knees are some of the most frequently affected joints, resulting in the global prevalence of knee OA (KOA) ranging from 1 to 10% among adults [2, 3]. The degeneration of the joint cartilage and underlying bone leads to stiffness and dull aching upon movement, which progresses to pain and decreased range of motion [2]. KOA is responsible for 2% of years lived with disability [4], often results in early retirement [5], and, thus, is a growing public health issue.

No treatments have been found to slow, prevent, or reverse KOA progression. The mainstay of treatments for KOA primarily targets the symptomatic aspects of the disease. Nonsteroidal anti-inflammatory drugs (NSAIDs), lifestyle adjustments, and intra-articular (IA) injections of corticosteroids or hyaluronic acid (HA) have shown efficacy for short-term symptomatic relief [5–8].

To conduct a scientifically robust clinical trial, the methodology and metrology are of great importance. For OA, the Osteoarthritis Research Society International (OARSI) established a task force in 1996 to help parties involved in the design, conduct, and reporting of clinical trials in OA [9]. In 2015, a working group updated these recommendations and established guidelines for clinical trials that target symptom or structure modification among individuals with KOA. The document serves as an important and tailored guideline providing strategies to address the heterogeneity of KOA and includes 25 recommendations regarding randomization, blocking and stratification, blinding, enhancing the accuracy of patient-reported outcomes (PRO), selecting a study population and index knee, describing interventions, patient-reported and physical performance measures, structural outcome measures, biochemical biomarkers, and reporting recommendations [10].

This study assessed the OARSI recommendations on clinical trials published between 2010 and 2020 investigating the efficacy or safety IA interventions in patients with KOA via a targeted literature review. The purpose of this study was to determine the impact of the OARSI recommendations on the design, conduct, and reporting of clinical trials after, as compared to before, the publication of the OARSI recommendations in 2015.

## Methods

### Search strategy

Relevant studies were identified on January 13, 2021, by searching the Cochrane Controlled Register of Trials (via Ovid) for publications of randomized controlled trials (RCTs) in KOA that were published between 2010 and 2020 inclusive (available in Additional file 1).

### Study selection and extraction

The study eligibility criteria were defined using the Population, Intervention, Comparator, and Outcome (PICO) framework. RCTs that targeted symptom or structure modification and investigated intra-articular therapies in adult patients with KOA were included. Only English-language publications and studies published between 2010 and 2020 were included. Eligible studies were included for data extraction and assessment using the OARSI recommendations.

### Assessment of clinical trials

The quality of study conduct, design, and reporting of eligible trials was assessed using the OARSI clinical trials recommendations [10], based on published journal articles and additional online trial registry records and study protocols when available. The authors assessed the trials independently of each other. Each OARSI recommendation was assessed as either “yes”, “no”, “unclear”, or “not applicable” as to whether the recommendation was addressed. Recommendations that were deemed as either “yes” or “not applicable” were considered to be adequately addressed or adhered to. Additionally, the 25 recommendations were categorized into 18 domains, as outlined in the OARSI guideline [10]. An overview of all 25 OARSI recommendations and 18 domains is depicted in Table 1.

**Table 1:** OARSI clinical trials recommendations [10]

Domain	Recommendations
Randomization	1. Trial methodology includes effective randomization procedures that ensure that members of the study team and participants remain unable to predict or influence their treatment assignment.
Blocking and stratification	2. Any stratification and/or subset analyses are specified prior to study development.
Blinding	3. Adequate blinding procedures are used to prevent disclosure of allocation to participants and study staff.
	4. If adequate blinding is not possible, an independent staff member, ideally not aware of the study hypotheses, performs the assessments or procedures that may lead to disclosure of allocation assignment (e.g., injections or exercise intervention).
	5. It is clearly indicated who was blinded and the mechanisms by which this was accomplished are described.
Expectations	6. Research staff is trained about the importance of equipoise when discussing the study interventions with the participants.
Washout periods and concomitant pain medications	7. Design strategies are adopted to manage confounding by concomitant medications.
Outcome reporting training	8. Steps are taken to ensure consistency and accuracy of outcome reporting by participants.
Comorbidities and subphenotypes	9. Study design is explicit about inclusion or exclusion of comorbidities.
	10. Planning is conducted for recruiting or analyzing sub-phenotypes.
Characterizing baseline disease	11. The severity of the disease, the pathological sub-phenotype (e.g., synovitic, bone marrow lesion, meniscal), structural sub-phenotype (e.g., knee compartment), and pain sub-phenotype (e.g., neuropathic, nociceptive) are characterized at baseline
Selecting an index knee	12. If selection of an index knee is required, selection strategy is defined in advance.
Symptom-modifying interventions	13. Symptomatic cut-points are selected to avoid ceiling or floor effects and permit analysis of the minimally clinically important differences.
Structure-modifying interventions	14. Structural severity cut-off points are selected to avoid ceiling or floor effects and permit analysis of the minimally clinically important differences.
Trial interventions	15. Interventions (active and placebo) are described in sufficient detail to allow others to replicate them.
Trial outcome measures	16. Trials use patient-reported and objective outcome measures that are valid, reliable, and responsive to change.
	17. Primary and secondary outcome measures are defined a priori and indicated when registering a trial.
Patient-reported outcome measures	18. Symptomatic outcomes are assessed using the three core clinical measures: pain, physical function, and patient global assessment.
Objective outcome measures (e.g., physical function)	19. A set of physical performance measures for knee OA are used.
Structural outcome measures	20. Radiography or MRI are used for demonstration of structure modification. The choice of imaging technique and outcome measures (primary and secondary) should be predicated on the expected mechanism of the intervention (e.g., synovitis/effusion volume for anti-inflammatory agents)
	21. Reliability and other metrics of measurement error and sensitivity, including scan-rescan reproducibility, are assessed at each study site.
	22. Disease modification is defined as an improvement in KOA-related symptoms (e.g., joint pain) and one of the following structural outcomes: reduction or reversal of joint space narrowing (continuous outcome); or reducing the progression of cartilage damage or reversal of cartilage damage on MRI (e.g., thickness, denudation).
Biochemical biomarkers	23. Biological fluids are collected and stored to assess the metabolic effect of a treatment on joint tissues.
Reporting	24. The clinical trial is registered in the appropriate registry prior to enrolling participants (e.g., Clinicaltrials.gov).
	25. The clinical trial methodology and results are reported in a format that allows for their inclusion in pooled analyses.

### Adherence to OARSI recommendations

Adherence was assessed as the number of OARSI recommendations that were completely addressed for each clinical trial. To compare adherence to the OARSI recommendations before and after the 2015 update, the year 2016 was selected as the cut-off to accommodate time for the uptake of the published guideline.

Furthermore, studies were categorized as having low or high adherence to the OARSI recommendations. The decision for a cut-off value was data-driven and based on the median number of OARSI recommendations that were adequately addressed across all clinical trials. Low adherence was defined as addressing less than the median number of recommendations and high adherence was defined as addressing the median number of recommendations or more. Finally, a select number of publications were randomly chosen and described in greater detail as case studies of publications with low and high adherence.

## Results

### Study selection

**Fig. 1:**
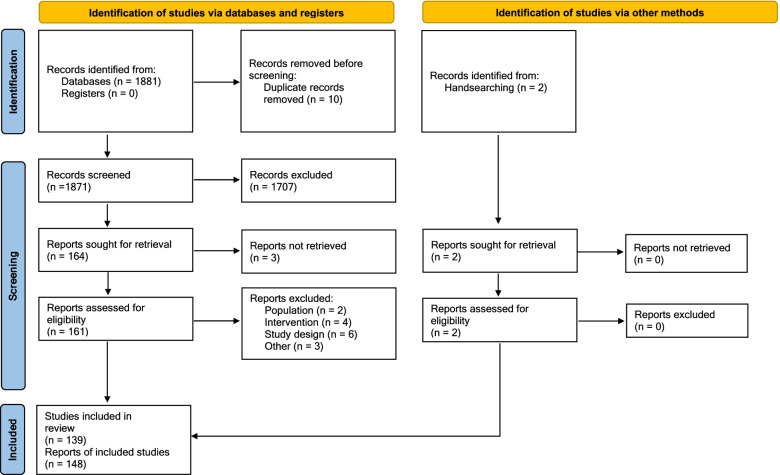
PRISMA flow diagram

After screening 1873 titles and abstracts, 166 full-text articles were reviewed for eligibility. Of these, 148 publications (139 unique trials) were included in this review Fig. 1.

### Trial characteristics

Of the 139 unique trials, 74 were published in 2016 or earlier and 65 were published after 2016 (available in Additional file 2). 109 trials started enrollment in 2016 or earlier and 30 did not specify the enrollment year. Of the studies that did not specify the enrollment year, 23 were published in 2016 or earlier, and 7 were published after 2016.

Most studies were conducted in Asia (*n* = 46), followed by Europe (*n* = 33), North America (*n* = 25), South America (*n* = 9), Australia (*n* = 2), and Africa (*n* = 1). Other trials were conducted multi-nationally (*n* = 21), or the study location was not reported (*n* = 2). In terms of blinding, 89 double-blind, 30 open-label, and 13 single-blind studies were included. Four studies did not report blinding procedures. The sample size ranged from 16 to 549 (median = 80), and the study follow-up ranged from 4 to 390 (median = 26) weeks across trials.

IA therapies included HA (*n* = 80 studies), corticosteroids (*n* = 29), platelet-rich plasma (*n* = 25), mesenchymal stem cells (*n* = 19), botulinum toxin A (*n* = 6), anesthetics (*n* = 5), NSAIDs (*n* = 3), ozone (*n* = 3), polynucleotides (*n* = 3), sprifermin (*n* = 3), TissueGene-C (*n* = 3), albumin (*n* = 2), and dextrose (*n* = 2). Other IA therapies were adalimumab, tropomyosin receptor kinase A inhibitor, clodronate, collagen, sodium bicarbonate, calcium gluconate, BIOF2 (a mixture of corticosteroid, insulin, and organic acids), and recombinant human bone morphogenetic protein (BMP-7).

### Assessment of OARSI guidelines

**Fig. 2:**
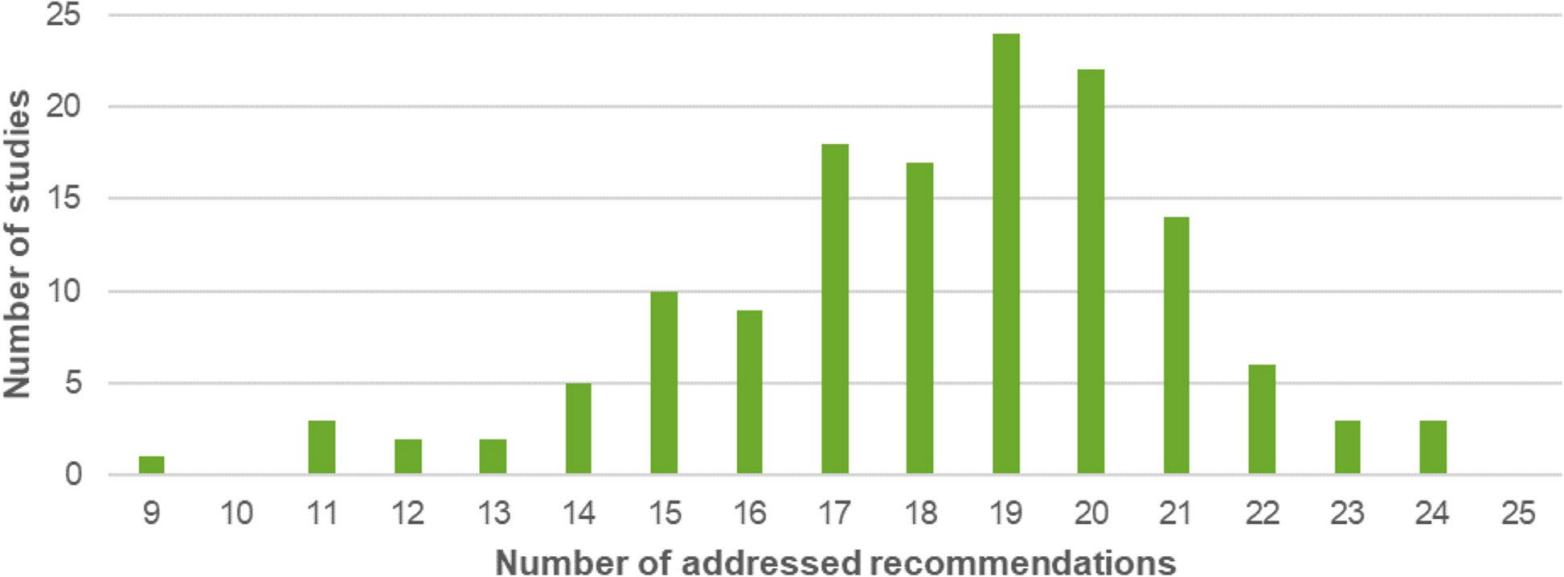
Distribution of the number of addressed OARSI recommendations across trials (max. Number of 25 recommendations; median was 19 recommendations)

Included studies adhered to between 9 and 24 (median: 19) of the 25 OARSI recommendations. The distribution of the number of addressed OARSI recommendations across trials is illustrated in Fig. 2.

#### Trial design domains: recommendations #1-#8

The majority of studies included effective randomization procedures (recommendation #1; *n* = 124; 89.2%), specified stratification or subset analyses a priori when applicable (recommendation #2; *n* = 132; 95.0%), included adequate blinding procedures (recommendations #3-#5; *n* = 90; 64.7%), adopted strategies to manage confounding by concomitant medications (recommendation #7; *n* = 115; 82.7%), and ensured consistency and accuracy of outcome reporting by participants (recommendation #8; *n* = 138; 99.3%). None of the studies reported the training of research staff regarding the importance of equipoise when discussing the study with participants (recommendation #6).

#### Study sample and patient selection domains: recommendations #9-#14

Most studies were explicit in terms of the inclusion of comorbidities or subphenotypes (recommendations #9-#10; *n* = 132; 95.0%), characterized KOA severity at baseline (recommendation #11; *n* = 124; 89.2%), and reported structural severity cut-points, such as a minimum Kellgren-Lawrence grade, for the inclusion of participants (recommendation #14; *n* = 125; 89.9%). Less studies adhered to the recommendation for symptom-modifying interventions (recommendation #13), as merely 69.8% of studies (*n* = 97) used symptomatic cut-points for the inclusion of study participants. These cut-points are recommended to avoid ceiling or floor effects of an intervention. For example, one study only included KOA patients with a baseline knee pain score of ≥30 mm on a 100-mm visual analog scale (VAS) [11], while another used a score of ≥4 on a 10-point numeric scale for the inclusion of patients [12]. Using these cut-points ensures that patients experience sufficient pain or other symptoms to demonstrate a potential symptom-modifying effect of the intervention. Additionally, lower adherence rates were seen for selecting an index knee (recommendation #12; *n* = 89; 64.0%), as not all studies defined a strategy for the selection of the target knee when applicable.

#### Trial interventions domain: recommendation #15

Most studies (*n* = 135; 97.1%) described the trial interventions (recommendation #15) in sufficient detail to allow replication of the study. Intervention details included its dose and frequency of administration.

#### Outcome measures domains: recommendations #16-#23

Most studies used and defined valid and reliable primary and secondary outcomes, addressing the trial outcome measures recommendations (recommendations #16-#17; *n* = 111; 79.9%), and assessed PROs regarding pain, function, and patient global assessment (recommendation #18; *n* = 134; 96.4%). However, fewer studies used objective outcome measures (recommendation #19; *n* = 22; 15.8%) or used magnetic resonance imaging (MRI) or radiography to demonstrate structure modification (recommendations #20-#22; *n* = 50; 36.0%). Examples of structure modification measures that were reported in studies included changes in joint space width [13–16] and articular cartilage volume [14, 17, 18]. Another recommendation with low adherence was the recommendation for the use of biochemical biomarkers (recommendation #23). In total, 22 studies (15.8%) collected and stored biological fluids, such as blood or synovial fluid, to assess the metabolic effect of an intervention on joint tissues. Collected biomarkers varied across studies, and included inflammatory biomarkers (e.g., cytokines and chemokines), collagenous biomarkers, non-collagenous biomarkers (e.g., cartilage oligomeric matrix protein and hyaluronan), and others.

#### Trial registration and reporting domains: recommendations #24-#25

All but one of the studies sufficiently reported clinical trial methodology and results in a format that allows for their inclusion in pooled analyses (recommendation #25; *n* = 138; 99.3%). However, not all studies reported registering the clinical trial in an appropriate trial registry or registered the clinical trial prior to enrollment (recommendation #24; *n* = 53; 38.1%).

#### Comparison between time periods

**Fig. 3:**
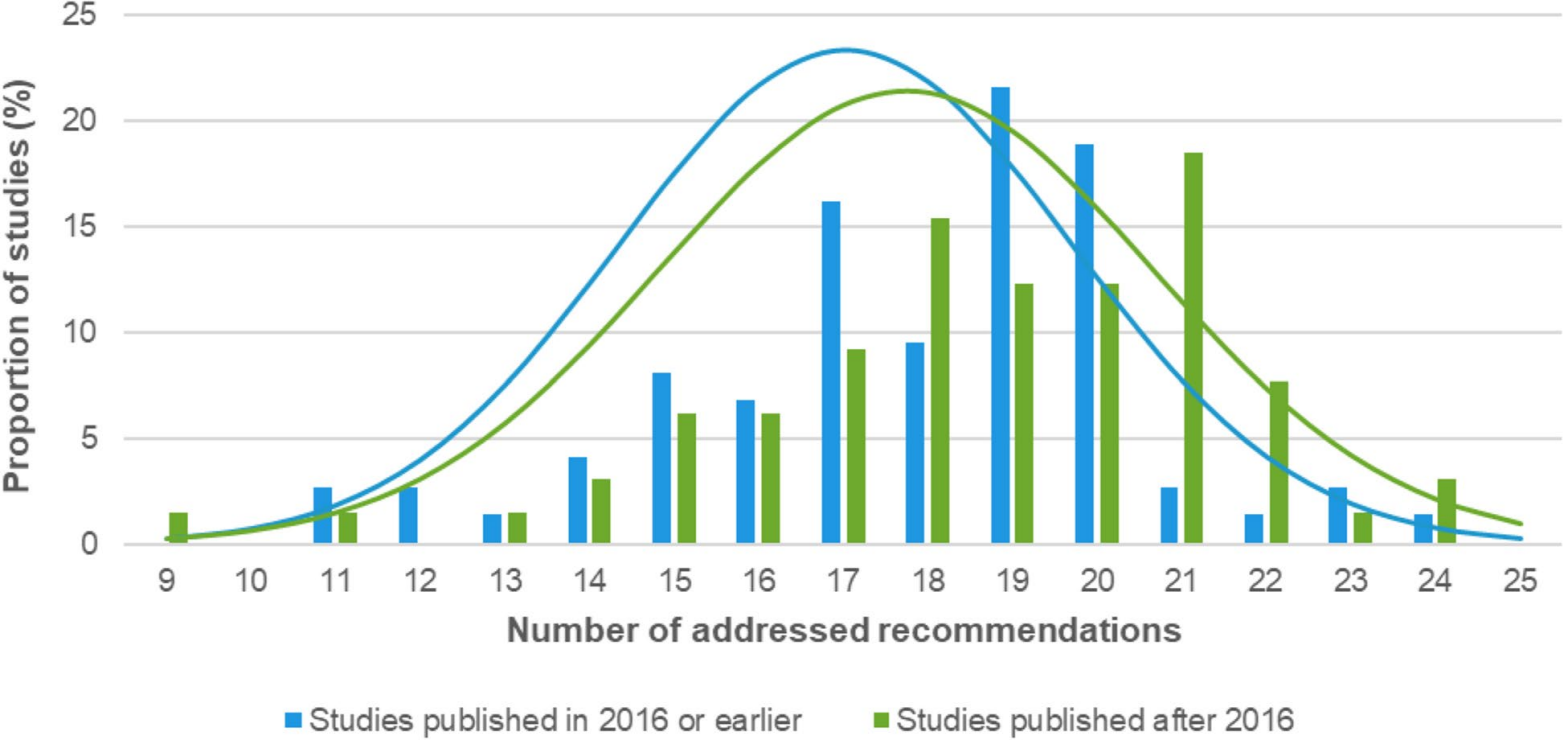
Distribution of the number of addressed OARSI recommendations across trials (max. Number of 25 recommendations), stratified by publication year

Overall, the adherence of studies published after 2016 (*n* = 65), compared to 2016 or earlier (*n* = 74), was trend-wise higher. Studies published in 2016 or earlier adhered to a median number of 18 recommendations (range: 11 to 24), while studies published after 2016 adhered to a median number of 19 recommendations (range: 9 to 24). The distribution of the number of addressed OARSI recommendations stratified by publication year can be seen in Fig. 3.

**Fig. 4:**
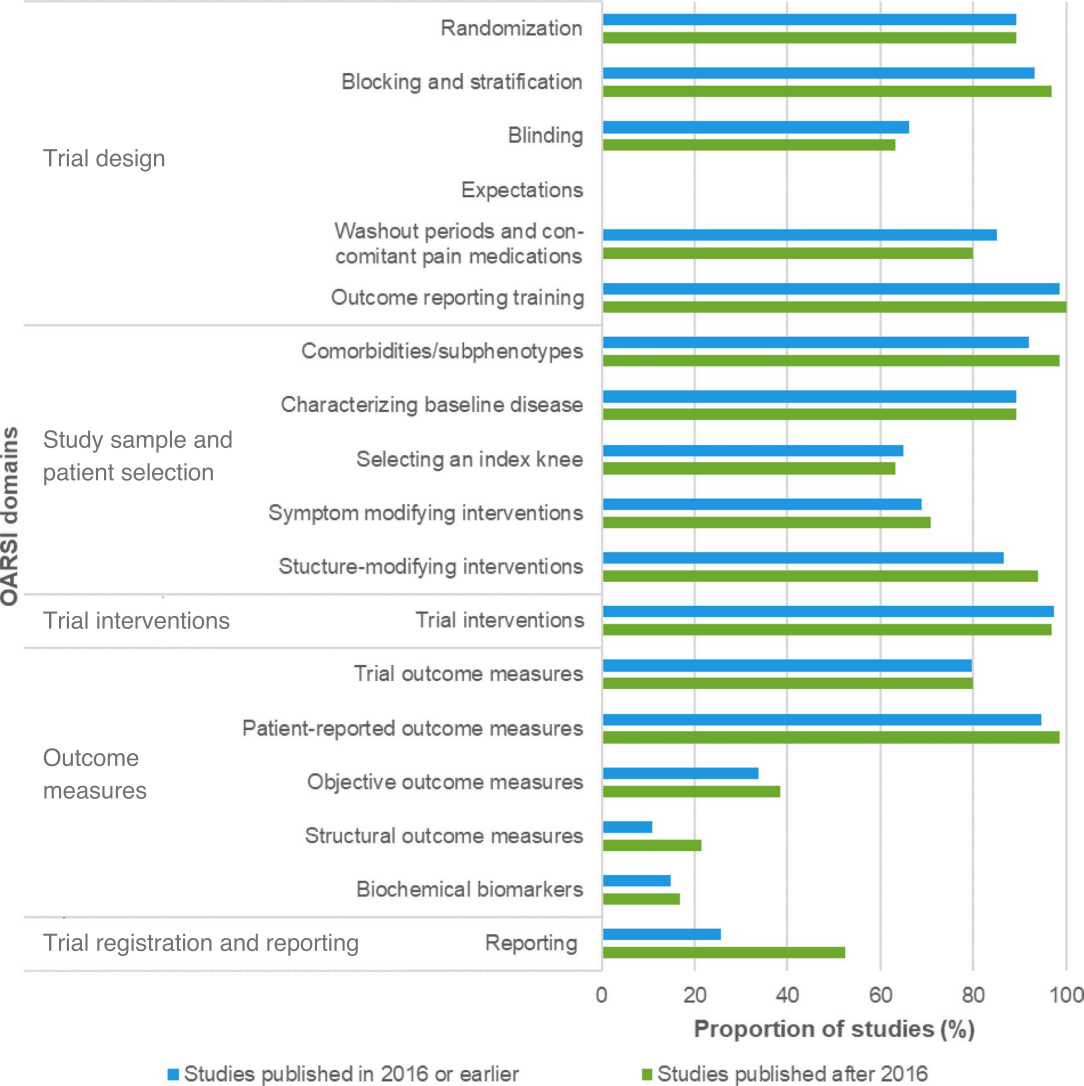
Proportion of studies that fully addressed the OARSI recommendations per domain, stratified by publication year

Adherence to the OARSI recommendations was compared between time periods per each of the 18 domains, consisting of multiple recommendations. The domain with the highest increase in adherence from studies published after 2016 compared with 2016 or earlier was the reporting domain (recommendations #24-#25; 25.7 to 52.3%), followed by the structural outcome measures (recommendations #20-#22; 10.8 to 21.5%), structure-modifying interventions (recommendation #14; 86.5 to 93.8%), and comorbidities/subphenotypes domains (recommendations #9-#10; 91.9 to 98.5%). The proportion of studies within each time period that fully addressed the OARSI recommendations per domain, stratified by publication year, is depicted in Fig. 4.

**Fig. 5:**
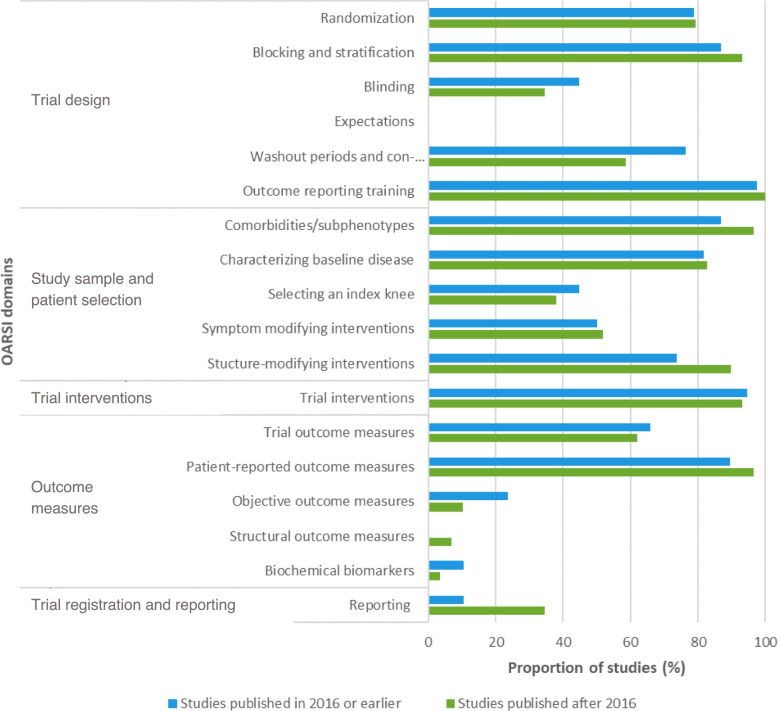
Proportion of low adherence studies that fully addressed the OARSI recommendations per domain, stratified by publication year

Overall, 67 studies (48.2%) had low adherence to the OARSI recommendations based on our definitions (i.e., adhered to < 19 recommendations), whereas 72 studies (51.8%) had high adherence. Across low adherence studies, an increase in adherence to the recommendations of the reporting domain (recommendations #24-#25; 10.5 to 32.1%) was seen for studies published after 2016 compared with 2016 or earlier. However, a decline in adherence was also seen for certain domains, including the washout periods and concomitant pain medications (recommendation #7; 76.3 to 57.1%), objective outcome measures (recommendation #19; 23.7 to 10.7%), and blinding domains (recommendations #3-#5; 44.7 to 32.1%). The adherence rates for all domains of the OARSI guidelines are depicted in Fig. 5, which illustrates the proportion of low adherence studies that fully addressed the OARSI recommendations per domain, stratified by publication year (i.e., ≤ 2016 versus > 2016).

**Fig. 6:**
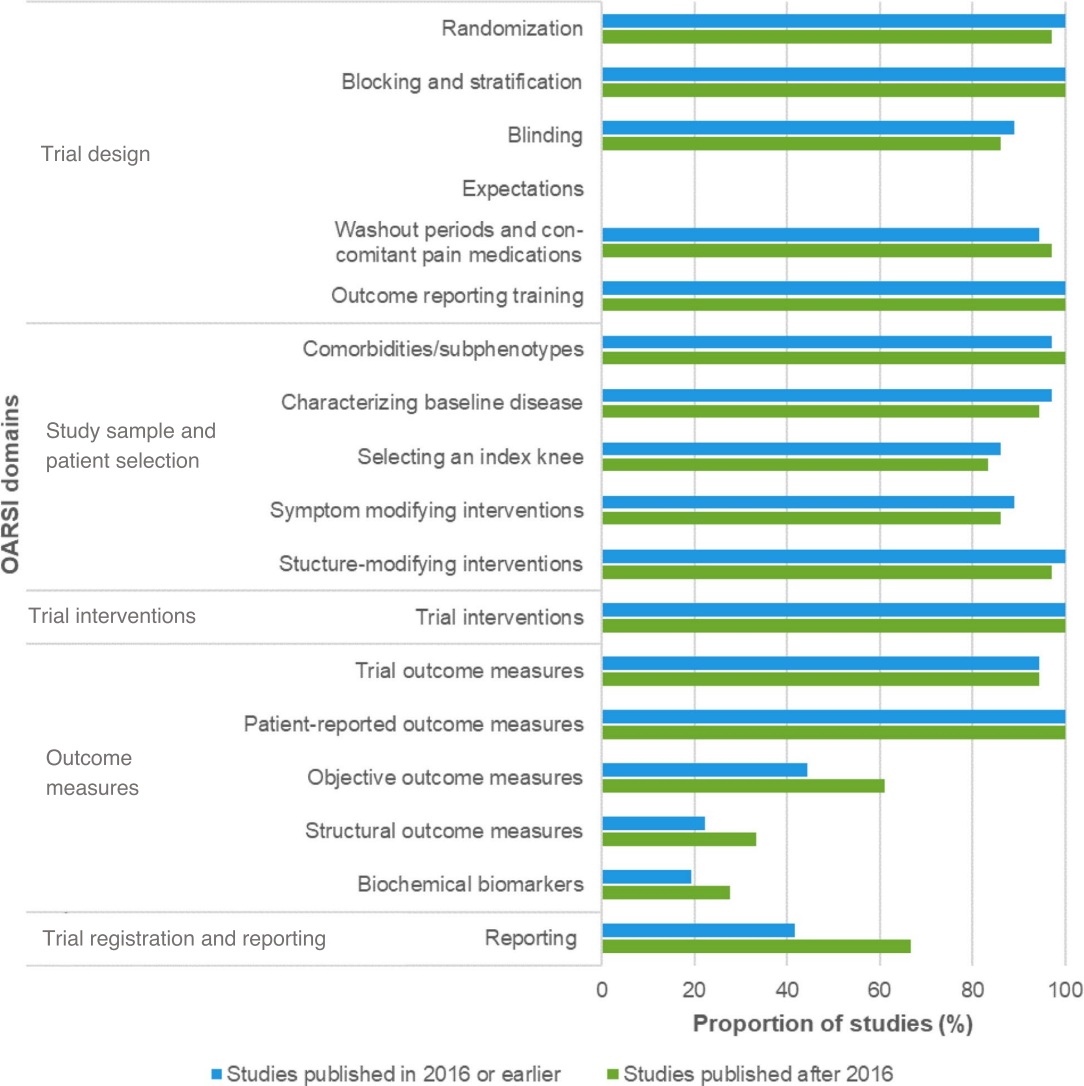
Proportion of high adherence studies that fully addressed the OARSI recommendations per domain, stratified by publication year

Across high adherence studies, the lowest adherence was seen for the trial registration and reporting domain (recommendations #24-#25) for studies published in 2016 or earlier, while for studies published after 2016, the objective outcome measures (recommendation #19), structural outcome measures (recommendations #20-#22), and biochemical biomarkers (recommendation #23) domains were adhered to the least. Compared with studies published in 2016 or earlier, adherence increased across studies published after 2016, especially for the reporting (recommendation #24-#25; 41.7 to 67.6%), objective outcome measures (recommendation #19; 44.4 to 59.5%), structural outcome measures (recommendations #20-#22; 22.2 to 32.4%), and biochemical biomarkers domains (recommendation #23; 19.4 to 27.8%). However, for other domains, adherence remained the same or even declined (Fig. 6).

### Case studies

Two low adherence and two high adherence publications were selected as case studies for illustration [19–22]. Table 2 highlights the differences between these publications with regards to the OARSI domain assessments.

**Table 2:** Case study summary

Domain	Low adherence (addressed < 19 recommendations)		High adherence (addressed ≥ 19 recommendations)	
	McGrath, 2013	Al-Omran, 2014	McAlindon, 2017	Chevalier, 2010
**Randomization** (max. ✓)			✓	✓
**Blocking/stratification** (max. ✓)	✓	✓	✓	✓
**Blinding** (max. ✓✓✓)	✓	✓	✓✓✓	✓✓✓
**Expectations** (max. ✓)				
**Washout periods/concomitant medications** (max. ✓)	✓	✓	✓	✓
**Outcome reporting training** (max. ✓)	✓	✓	✓	✓
**Comorbidities/** **subphenotypes** (max. ✓✓)	✓✓	✓✓	✓✓	✓✓
**Characterizing baseline disease** (max. ✓)			✓	✓
**Selecting an index knee** (max. ✓)	✓		✓	
**Symptom-modifying interventions** (max. ✓)			✓	✓
**Structure-modifying interventions** (max. ✓)	✓	✓	✓	✓
**Trial interventions** (max. ✓)			✓	✓
**Trial outcome measures** (max. ✓✓)	✓	✓✓	✓✓	✓✓
**Patient-reported outcome measures** (max. ✓)	✓		✓	✓
**Objective outcome measures** (max. ✓)	✓		✓	
**Structural outcome measures** (max. ✓✓✓)	✓	✓	✓✓✓	✓
**Biochemical biomarkers** (max. ✓)			✓	
**Reporting** (max. ✓✓)		✓	✓✓	✓✓
**Total number of addressed recommendations**	**12**	**11**	**24**	**19**

✓ = Recommendation was adequately addressed in the trial publication or was not applicable

#### Low adherence studies

The study by Al-Omran et al. (2014) randomized 227 patients to one of three HA products: Osteonil®, Durolane®, or Synvisc® [21]. The authors concluded that all three agents provide significant symptomatic relief with “marginal superiority” of Synvisc over the other two products. The study was not sponsored, and the authors declared no competing interest or potential conflicts of interest. This study addressed only 11 OARSI recommendations, as several methodological and reporting deficiencies were identified. First, recommendations #1, #3, and #5 were not addressed as their randomization and blinding procedures were not reported. Recommendations #11 and #12 were also not addressed as the authors did not characterize the disease at baseline and did not report their strategy on selecting an index knee. Recommendation #13 was not addressed as no symptomatic cut-point was included in the study’s eligibility criteria. Additionally, the authors did not provide sufficient details on the interventions to permit others to replicate the study (recommendation #15). For example, details regarding the dose and frequency of administration were not provided. Recommendations #18, #19, #20, #22, and #23 were inadequately addressed, as the publication did not report on all three core clinical measures, did not report on an objective physical performance outcome measure, and did not measure any structural outcome measures or biochemical biomarkers. Lastly, the trial was not registered prior to enrollment (recommendation #24).

The study by McGrath et al. (2013) recruited 213 patients to receive either Durolane® or Synvisc® [19]. The authors concluded that significant differences were noted in efficacy and adverse reactions between the two interventions, favoring Durolane; however, the study also suffered from several limitations and addressed only 12 recommendations. First, randomization procedures were not reported (recommendation #1), and it is unclear how many patients were randomized and evaluated. Authors stated that 213 patients were recruited and a final sample size of 168 (a difference of 45 patients); however, they reported only 31 patients as lost to follow-up, leaving 14 patients unaccounted for. Additionally, they did not characterize baseline disease (recommendation #11), did not include a symptomatic cut-point in their eligibility criteria (recommendation #13), and did not describe the trial interventions in sufficient detail to allow others to replicate this study (recommendation #15). Information on Durolane and Synvisc was provided in the introduction section of the article, where both were described as a single-injection therapy, but the authors did not provide further details for the specific dose and administration regimen used in this study. However, at one point, the authors described patients receiving Synvisc as the “Synvisc-3 group”, which implies three injections were given, leaving intervention details unclear. Furthermore, recommendations #3 and #5 were not addressed, as blinding procedures were not reported, and if single-injection Durolane was compared to 3-injection Synvisc, blinding may have been compromised. Recommendations #17, #20, #22, and #23 were also not addressed, as the primary and secondary outcomes were not defined, and structural outcomes and biochemical biomarkers were not measured. Lastly, recommendations #24 and #25 were not addressed as the trial was not registered prior to enrollment, and there was insufficient information for its inclusion in a pooled analysis. Baseline demographics, including the sample sizes, were not described, and the number of adverse events was not reported separately for each intervention. Of note, study sponsorship and potential conflicts of interest were not disclosed.

#### High adherence studies

Chevalier et al. (2010) randomized 253 patients to either Synvisc or placebo [22]. The study was sponsored by the intervention’s manufacturer. The authors concluded that Synvisc is safe and effective in providing statistically significant and clinically relevant pain relief over 26 weeks. The study adhered to 19 recommendations and failed to address only six OARSI recommendations. Reasons for not adhering to all recommendations include that the authors did not define their strategy for selecting the index knee (recommendation #12), and did not measure an objective physical performance outcome (recommendation #19), structural outcome (recommendations #20-#22), or biochemical biomarkers (recommendation #23).

In a 2017 McAlindon et al. publication, the authors conducted a trial evaluating IA-corticosteroid (triamcinolone) versus placebo [20]. A total of 140 patients were randomized and followed for 2 years. The study was not industry-sponsored, and the intervention was purchased from the manufacturer. This study failed to address only one OARSI recommendation, as the authors did not report the training of research staff regarding the importance of equipoise when discussing the study interventions with patients (recommendation #6), resulting in 24 addressed recommendations.

These case studies further exemplify the methodological and reporting differences between trials that were less versus more adherent to the OARSI recommendations. Though these studies all report on interventions within the same treatment category (i.e., IA injections), or even within the same treatment brand (e.g., Synvisc), clear differences are seen.

## Discussion

The current study assessed the adherence of clinical trials published between 2010 and 2020 investigating IA interventions in patients with KOA to the 2015 OARSI recommendations. A slight increase in adherence for studies published after the introduction of the 2015 OARSI recommendations was observed. The highest increase in adherence was seen in the reporting and registration of the trial, and the use of structural outcome measures. Despite an overall increase in adherence after the introduction of the new OARSI recommendations, adherence remained low in terms of training research staff about the importance of equipoise when discussing treatments with study participants, the measurement of biochemical biomarkers, and the use of structural outcome measures.

These findings suggest that there has been a slight improvement in the design, conduct, and reporting of clinical trials investigating the efficacy or safety of IA interventions for KOA over time in the last decade. After the introduction of the new OARSI recommendations, more trials were registered prior to study enrollment, more trials collected biological fluids of patients to assess the metabolic effects of interventions on joint tissues, and more trials measured and reported on using MRI or radiography to demonstrate structure modification in the knee joint. Lastly, more trials reported on patient comorbidities and subphenotypes, which could help clinicians identify appropriate patient types for different treatments [23]. The design, conduct, and reporting of these trials could be improved further by evaluating physical performance measures for KOA, defining strategies for the selection of the index knee (when applicable), and using and reporting adequate blinding procedures. Adequate blinding is especially important in KOA research, as outcomes such as pain and disability are subjective and patient-reported [24]. However, as illustrated with the four case studies [19–22], all methodological decisions can influence the interpretation of study results. For instance, the studies by McGrath et al. [19] and Al-Omran et al. [21] both compared Durolane and Synvisc, but reported contradicting results, which may be due partially to differences in the conduct and design of the trials, with McGrath et al. describing a greater improvement with Durolane, while Al-Omran et al. reported a superior effect of Synvisc. Thus, limitations in the conduct and reporting of clinical trials can lead to biased results and should be prevented to the fullest extent possible in order to compare between studies evaluating the same treatment comparison more accurately (i.e., methodological heterogeneity) [25–27].

To the best of our knowledge, no prior studies have been conducted on the quality assessment of KOA trials on IA injections using the OARSI recommendations; however, similar studies using other study quality and reporting guidelines exist [24, 25, 28, 29]. Chan et al. (2021) showed that the use of different guidelines to assess clinical trial quality can lead to different quality scoring, using the Oxford Levels of Evidence [30], a modified Coleman Methodology Score [31], and the revised Consolidated Standards of Reporting Trials (CONSORT) score [32]. Although their study included only clinical trials on platelet-rich plasma, similar methodological weaknesses of clinical trials were found, including the measurement of clinical effects, blinding, allocation concealment, and the implementation of randomization [28]. Boutron et al. (2003) reported that the overall quality of clinical trials on nonpharmacological and pharmacological interventions for hip and knee OA was poor. Quality was assessed using the Jadad scale [33] and the Delphi list [34] and, similarly, the greatest methodological deficiencies found were the lack of blinding and allocation concealment, and inadequate randomization procedures [24]. This was confirmed by a study by Hill et al. (2002), who assessed the quality of RCTs in rheumatology between 1987 and 1988 and 1997–1998 [29]. Similarly, the authors reported an improvement in the overall quality of RCTs over time, despite remaining deficiencies.

A deficiency across clinical trials that was found in the current review was a lack of reporting on biochemical biomarkers. Specific biomarkers related to metabolic changes due to KOA may be used for monitoring its progression, as well as for treatment development [35, 36]. This is a topic that is gaining interest in KOA research, especially since current trials focus mostly on PROs and are unable to show metabolic changes of therapies on joint tissues. However, there is currently no consensus on the collection of a specific set of biomarkers, as only a small number of biomarkers involved in the development of KOA have been identified so far [10, 36–38]. For example, Yang et al. (2015) showed that treatment with HA injections significantly reduced the concentration of cytokine interleukin-1β (IL-1β) in synovial fluid [39], while a study by Henrotin et al. (2017) reported decreased levels of serum Coll2–1 [40]. Both IL-1β and Coll2–1 are biomarkers of cartilage degradation in KOA and could be potentially used to assess treatment effects of HA injections [39, 40]. Despite the overall variability of reported biomarkers and their metabolic effect on joint tissues, collecting biological fluids is of importance in order to qualify a biomarker or a cluster of biomarkers for the development of drugs for KOA treatment [10].

The current study used the OARSI recommendations, which is a recently published guideline developed specifically for the assessment of KOA trials and takes into consideration unique challenges in this field; however, a limitation of the current study was that by using only one set of recommendations, important criteria may have been missed that are included in other guidelines, such as the inclusion of an intention-to-treat (ITT) analysis [34], study sample size and follow-up [31, 33], and funding [32]. Secondly, a lack of reporting does not necessarily mean that certain procedures were not done properly; the fault might have been in the reporting only, not in the conduct or design of the trial [24, 29]. This may explain the lack of reporting for recommendation #6 (training staff in equipoise). Thirdly, this study was a targeted literature review with the intention to provide a narrative summary of the most relevant findings related to the stated objectives of the study without providing additional statistical analyses. As well, only one database was searched, thus some relevant studies might have been missed if they are not indexed in this particular database. Finally, the impact of the introduction of the new OARSI recommendations in 2015 on trial quality remains unclear. Although all included trials were published between 2010 to 2020, most were conducted prior to 2016. More time may be needed to assess the uptake of the OARSI recommendations more accurately among KOA investigators. A lack of awareness and consensus on guidelines such as the OARSI may also explain the wide range of adherence levels, especially for certain recommendations that consistently demonstrated low adherence; therefore, broad dissemination and acceptance of such guidance is a common goal that the KOA research community should strive to achieve.

While the OARSI guidelines do refer readers to the CONSORT statement for general design of clinical trials, future updates to the recommendations in KOA trials could make considerations to include the more general, yet still important, criteria in trial design. For example, the ITT approach analyzes patients according to the group they were originally assigned in order to preserve the prognostic balance between treatment groups [41]. This is an important consideration in RCTs as deviations from this method of analysis may result in biased and inaccurate estimates of comparative treatment effects. The study’s sample size is also an important factor when planning a RCT as the results of a trial need to be interpreted within the context of a number of statistical parameters (e.g., statistical power, alpha level, etc.) [32]; therefore, how the study investigators determined the final sample size should also be reported. Funding or sponsorship is another criterion that may be added to the OARSI recommendations, as this can introduce bias into many aspects of a clinical trial’s design and reporting [42].

## Conclusion

This study suggests that an improvement can be made in the conduct, design, and reporting of clinical trials for IA therapies in KOA. Despite proper guidelines, the trial quality varies, and the methodological deficiencies found are preventable and can be corrected. Continued efforts are needed to improve the quality of KOA trials with a focus on the use of biochemical biomarkers and reporting of comorbidities and KOA subphenotypes. This will allow improved comparison and consistency between trials and will in turn allow clinicians to interpret the literature with more certainty, leading to better treatment decisions for patients with KOA in clinical practice. Lastly, this study may provide some insight for future modifications to the OARSI guidelines themselves.

## Supplementary Information

**Additional file 1.** Search strategy for CENTRAL via OvidSP.

**Additional file 2.** Trial characteristics of included clinical trials.

## Data Availability

Not applicable. The data for this literature review was retrieved from published studies listed in the manuscript.

## Abbreviations

CONSORT: Consolidated Standards of Reporting Trials
HA: Hyaluronic acid
IA: Intra-articular
KOA: Knee osteoarthritis
MRI: Magnetic resonance imaging
NSAIDs: Nonsteroidal anti-inflammatory drugs
OA: Osteoarthritis
OARSI: Osteoarthritis Research Society International
PICO: Population, intervention, comparator, and outcome
PRISMA: Preferred Reporting Items for Systematic Reviews and Meta-Analyses
PRO: Patient-reported outcomes
RCTs: Randomized controlled trials
