# Supplementary material for: Adherence to the OARSI recommendations for designing, conducting, and reporting of clinical trials in knee osteoarthritis: a targeted literature review
Source: BMC Musculoskelet Disord. 2022 Feb 22;23:171. doi: 10.1186/s12891-022-05116-z (PMC8864780; doi:10.1186/s12891-022-05116-z)
Supplement: Supplementary file 1 — Additional file 1. Search strategy for CENTRAL via OvidSP. [file 12891_2022_5116_MOESM1_ESM.docx]

Additional File 1 – Search strategy for CENTRAL via OvidSP

| **Database: EBM Reviews - Cochrane Central Register of Controlled Trials**  **Search executed: January 13, 2021** | | |
| --- | --- | --- |
| **#** | **String** | **Hits** |
| 1 | exp Osteoarthritis, Knee/ | 4346 |
| 2 | limit 1 to yr="2010 -Current" | 3263 |
| 3 | limit 2 to english | 1881 |
