# Supplementary material for: Adherence to the OARSI recommendations for designing, conducting, and reporting of clinical trials in knee osteoarthritis: a targeted literature review
Source: BMC Musculoskelet Disord. 2022 Feb 22;23:171. doi: 10.1186/s12891-022-05116-z (PMC8864780; doi:10.1186/s12891-022-05116-z)
Supplement: Supplementary file 2 — Additional file 2. Trial characteristics of included clinical trials. [file 12891_2022_5116_MOESM2_ESM.docx]

Additional File 2 – Trial characteristics of included clinical trials

| **Author and Publication Year** | **Study year** | **Study location/ country** | **Blinding** | **Sample size** | **Follow-up period, weeks** | **Treatment class** |
| --- | --- | --- | --- | --- | --- | --- |
| Ahmad et al 2018 | 2016-2017 | Egypt | Single-blind | 89 | 26 | Platelet-rich plasma vs. Hyaluronic acid |
| Al-Omran et al 2014 | NR | Saudi Arabia | Open-label | 227 | 26 | Hyaluronic acid (Osteonil) vs. Hyaluronic acid (Durolane) vs. Hyaluronic acid (Synvisc) |
| Arden et al 2014 | 2003-2004 | Multinational: Sweden, Germany, UK | Double-blind | 218 | 6 | Hyaluronic acid (Durolane) vs. Placebo |
| Arendt-Nielsen et al 2017 | 2012-2013 | Denmark | Double-blind | 121 | 12 | Botulinum toxin type A (Botox) vs. Placebo |
| Babaei-Ghazani et al 2018 | 2016-2017 | Iran | Double-blind | 62 | 13 | Corticosteroid vs. Ozone |
| Bao et al 2018 | 2016-2017 | China | Single-blind | 60 | 8 | Placebo vs. Botulinum toxin type A (Botox) vs. Hyaluronic acid |
| Bar-Or et al 2014 | 2013 | USA | Double-blind | 329 | 12 | LMWF-5A 4 ml (Ampion) vs. Placebo 4 ml vs. LMWF-5A 10 ml (Ampion) vs. Placebo 10 ml |
| Bastos et al 2018; Bastos et al 2020 | NR | Brazil | Double-blind | 18 | 52 | Mesenchymal stem cells vs. Mesenchymal stem cells + Platelet-rich plasma |
| Bellamy et al 2016 | 2014 | USA | Double-blind | 35 | 24 | IA NSAIDs (Toradol) vs. Corticosteroid (Kenalog) |
| Berenbaum et al 2012 | 2008-2009 | Multinational: France, Germany, Italy | Double-blind | 437 | 26 | Hyaluronic acid (GO-ON) vs. Hyaluronic acid (Hyalgan) |
| Bisicchia et al 2016 | NR | Italy | Single-blind | 150 | 52 | Hyaluronic acid (HYADD 4) vs. Corticosteroid (Depo-Medrol) |
| Bodick et al 2015 | 2012-2013 | Multinational: USA, Canada, Australia | Double-blind | 228 | 12 | Corticosteroid (FX006) vs. Corticosteroid (Kenalog/Kenacort) |
| Boon et al 2010 | NR | USA | Double-blind | 60 | 8 | Corticosteroid vs. Botulinum toxin type A 100 U (Botox) vs. Botulinum toxin type A 200 U (Botox) |
| Campos et al 2017 | 2015-2017 | Brazil | Double-blind | 120 | 26 | Corticosteroid vs. Hyaluronic acid (Synvisc) vs. Corticosteroid + Hyaluronic acid (Synvisc) |
| Centeno et al 2018 | 2014-2018 | USA | Open-label | 48 | 104 | Bone marrow aspirate concentrate + Platelet-rich plasma vs. Non-injectable therapy (exercise) |
| Cerza et al 2012 | 2009-2010 | Italy | NR | 120 | 24 | Platelet-rich plasma vs. Hyaluronic acid (Hyalgan) |
| Chao et al 2010 | NR | USA | Single-Blind | 79 | 12 | Corticosteroid vs. Placebo |
| Chen et al 2015 | 2011-2013 | China | Open-label | 432 | 26 | Hyaluronic acid (ARTZ) vs. Non-injectable therapy (transcutaneous electric nerve stimulation) |
| Chen et al 2013 | NR | Taiwan | Open-label | 54 | 13 | Non-injectable therapy (heat-sensitive moxibustion) vs. Non-injectable therapy (conventional moxibustion) vs. Hyaluronic acid (ARTZ) |
| Cherian et al 2015; Guermazi et al 2017 | 2011-2014 | USA | Double-blind | 102 | 52 | TissueGene-C vs. Placebo |
| Chevalier et al 2010 | 2005-2006 | Multinational: France, Germany, UK, The Netherlands, Belgium, Czech Republic | Double-blind | 253 | 26 | Hyaluronic acid (Synvisc One) vs. Placebo |
| Cole et al 2017 | 2011-2015 | USA | Triple-blind | 111 | 52 | Platelet-rich plasma vs. Hyaluronic acid (Synvisc) |
| Conaghan et al 2018 | 2014-2015 | Multinational: USA, Canada | Double-blind | 306 | 12 | Corticosteroid 16 mg (FX006) vs. Corticosteroid 32 mg (FX006) vs. Placebo |
| Conaghan et al 2018 | 2015-2016 | Multinational: France, UK, USA, Australia | Double-blind | 484 | 24 | Corticosteroid (FX006) vs. Placebo vs. Corticosteroid |
| Conrozier et al 2016 | 2012-2014 | France | Double-blind | 222 | 26 | Hyaluronic acid (HAppyVisc) vs. Hyaluronic acid (Euflexxa) |
| Dahlberg et al 2016 | 2007-2010 | Multinational: Sweden, Finland, Denmark, UK, Bulgaria, South Africa | Double-blind | 73 | 24 | Sprifermin (single ascending dose) vs. Sprifermin (multiple ascending dose) vs. Placebo |
| Dallari et al 2020 | 2014-2017 | Italy | Double-blind | 98 | 52 | Polynucleotides vs. Hyaluronic acid |
| Davis et al 2018 | 2015-2017 | USA | Open-label | 151 | 26 | Non-injectable therapy (RF neurotomy) vs. Corticosteroid (Depo-Medrol) |
| de Campos et al 2013 | NR | Brazil | Double-blind | 147 | 24 | Hyaluronic acid (Synvisc) vs. Hyaluronic acid + Corticosteroid |
| de Sire et al 2020 | NR | Italy | Single-blind | 42 | 31 | Ozone (Ozonline) vs. Hyaluronic acid (Hyalgan) |
| DeCaria et al 2012 | 2008-2009 | Canada | Single-blind | 30 | 26 | Hyaluronic acid (Suplasyn) vs. Placebo |
| Delgado-Enciso et al 2018 | 2015-2017 | Mexico | Open-label | 24 | 52 | BIOF2 vs. Non-injectable therapy (surgery) vs. Non-injectable therapy (oral NSAIDs) |
| Deyle et al 2020 | 2012-2018 | USA | Open-label | 156 | 52 | Corticosteroid vs. Non-injectable therapy (exercise) |
| Dracoglu et al 2016 | NR | Turkey | Open-label | 40 | 26 | Hyaluronic acid (Monovisc) vs. Hyaluronic acid (Adant) |
| Dumais et al 2012 | 2007-2008 | Canada | Open-label | 45 | 36 | Dextrose + Anesthetic vs. Non-injectable therapy (exercise) |
| Duymus et al 2017 | NR | Turkey | Open-label | 120 | 52 | Platelet-rich plasma vs. Hyaluronic acid vs. Ozone |
| Eker et al 2017 | 2009-2015 | Turkey | Double-blind | 52 | 13 | Anesthetic vs. Placebo |
| Elik et al 2020 | NR | Turkey | Double-blind | 60 | 26 | Platelet-rich plasma vs. Placebo |
| Emadedin et al 2018 | 2012-2015 | Iran | Triple-blind | 43 | 13 | Mesenchymal stem cells vs. Placebo |
| Erturk et al 2016 | 2009-2011 | Turkey | Open-label | 77 | 52 | Hyaluronic acid (Adant) vs. Hyaluronic acid + Corticosteroid + Anesthetic (Adant+Diprospan+Jetokain simplex) |
| Farr et al 2019 | 2015-2019 | USA | Single-blind | 200 | 13 | Amniotic suspension allograft (ReNu) vs. Hyaluronic acid (Monovisc) vs. Placebo |
| Filardo et al 2015; Di Martino et al 2019 | 2009-2014 | Italy | Double-blind | 192 | 52 | Platelet-rich plasma vs. Hyaluronic acid (Hyalubrix) |
| Filardo et al 2012 | NR | Italy | Double-blind | 109 | 52 | Platelet-rich plasma vs. Hyaluronic acid (Hyalubrix) |
| Forogh et al 2016 | NR | Iran | Double-blind | 41 | 26 | Platelet-rich plasma vs. Corticosteroid |
| Freitag et al 2019 | 2014-2018 | Australia | Open-label | 30 | 52 | Mesenchymal stem cells (single injection) vs. Mesenchymal stem cells (two injections) vs. Non-injectable therapy (exercise) |
| Garcia-Padilla et al 2015 | 2007-2008 | Mexico | Double-blind | 74 | 52 | Sodium bicarbonate and calcium gluconate 7.5% vs. Sodium bicarbonate and calcium gluconate 15% |
| Garza et al 2020 | 2016-2019 | USA | Double-blind | 39 | 26 | Stromal vascular fraction (high dose) vs. Stromal vascular fraction (low dose) vs. Placebo |
| Giarratana et al 2014 | NR | Italy | Double-blind | 72 | 26 | Polynucleotides vs. Hyaluronic acid (Hyalubrix) |
| Gobbi et al 2015 | 2009- | Italy | Open-label | 93 | 104 | Platelet-rich plasma (one cycle of 3 total doses) vs. Platelet-rich plasma (second cycle after 1 year) |
| Goncars et al 2017 | 2012-2015 | Latvia | Open-label | 56 | 52 | Bone marrow-derived mononuclear cells vs. Hyaluronic acid (GO-ON) |
| Gormeli et al 2017 | 2013 | Turkey | Double-blind | 182 | 26 | Platelet-rich plasma vs. Platelet-rich plasma + Placebo vs. Hyaluronic acid (Orthovisc) vs. Placebo |
| Guo et al 2018 | 2016-2017 | China | Double-blind | 258 | 26 | Hyaluronic acid (HYAJOINT Plus) vs. Hyaluronic acid (Synvisc) |
| Gupta et al 2016 | 2011-2013 | India | Double-blind | 60 | 104 | Mesenchymal stem cells (Stempeucel) vs. Placebo |
| Ha et al 2015 | 2009-2010 | Korea | Single-blind | 27 | 24 | TissueGene-C 6.0x10^6 cells vs. TissueGene-C 1.8x10^7 cells |
| Ha et al 2017 | 2011-2012 | Korea | Double-blind | 287 | 12 | Hyaluronic acid (LBSA0103) vs. Hyaluronic acid (Hyruan Plus) |
| Hangody et al 2018 | 2013-2014 | Multinational: Canada, Europe | Double-blind | 368 | 26 | Hyaluronic acid + Corticosteroid (Cingal) vs. Hyaluronic acid (Monovisc) vs. Placebo |
| Henriksen et al 2015; Soriano-Maldonado et al 2016; Riis et al 2017; Nielsen et al 2018 | 2012-2014 | Denmark | Double-blind | 100 | 26 | Corticosteroid (Depo-Medrol) + Non-injectable therapy (exercise) vs. Placebo + Non-injectable therapy (exercise) |
| Henrotin et al 2017 | 2013-2014 | NR | Double-blind | 81 | 26 | Hyaluronic acid (Kartilage Cross) vs. Placebo |
| Hermans et al 2019 | 2009-2012 | The Netherlands | Open-label | 156 | 52 | Hyaluronic acid (Synvisc) + Control vs. Control (usual care) |
| Hochberg et al 2019 | 2013-2017 | Multinational: Argentina, Czechia, Denmark, Estonia, Hong Kong, Poland, Romania, USA | Double-blind | 549 | 156 | Sprifermin 100 μg (every 6 months) vs. Sprifermin 100 μg (every 12 months) vs. Sprifermin 30 μg (every 6 months) vs. Sprifermin 30 μg (every 12 months) vs. Placebo |
| Hong et al 2019 | 2015-2018 | China | Double-blind | 16 | 52 | Stromal vascular fraction vs. Hyaluronic acid (Sofast) |
| Housman et al 2014 | 2004-2007 | Multinational: USA, Canada, France, UK, Germany | Double-blind | 391 | 26 | Hyaluronic acid vs. Hyaluronic acid vs. Corticosteroid |
| Hsieh et al 2016 | NR | Taiwan | Single-blind | 44 | 26 | Botulinum toxin type A (Botox) vs. Non-injectable therapy (education) |
| Huang et al 2011 | 2001-2002 | Taiwan | Double-blind | 200 | 25 | Hyaluronic acid (Hyalgan) vs. Placebo |
| Huang et al 2019 | 2016 | China | NR | 120 | 52 | Hyaluronic acid vs. Platelet-rich plasma vs. Corticosteroid |
| Hunter et al 2010 | 2007-2008 | USA | Double-blind | 33 | 24 | Recombinant human BMP-7 0.03 mg vs. Recombinant human BMP-7 0.1 mg vs. Recombinant human BMP-7 0.3 mg vs. Recombinant human BMP-7 1 mg vs. Placebo |
| Iannitti et al 2012 | NR | Italy | Triple-blind | 20 | 26 | Hyaluronic acid (Synvisc) vs. Hyaluronic acid + Corticosteroid (Variofill) |
| Ip and Fu 2015 | 2008-2015 | NR | Double-blind | 70 | 390 | Non-injectable therapy (exercise) + Placebo vs. Non-injectable therapy (exercise) + Hyaluronic acid (Hyalgan) + Non-injectable therapy (radiation) |
| Ishijima et al 2014 | 2008-2010 | Japan | Open-label | 200 | 5 | Hyaluronic acid (Suvenyl) vs. Non-injectable therapy (oral NSAIDs) (Roxonen) |
| Jorgensen et al 2010 | NR | Denmark | Double-blind | 337 | 52 | Hyaluronic acid (Hyalgan) vs. Placebo |
| Khalifeh Soltani et al 2019 | 2015-2016 | Iran | Double-blind | 20 | 24 | Mesenchymal stem cells vs. Placebo |
| Khanasuk et al 2012 | 2010-2011 | Thailand | Double-blind | 32 | 26 | Hyaluronic acid (Synvisc) vs. Hyaluronic acid (Hyalgan) |
| Kon et al 2018 | 2014-2016 | Multinational: Austria, Belgium, Italy, Norway | Double-blind | 46 | 52 | Autologous protein solution vs. Placebo |
| Krupka et al 2019 | 2016-2017 | Germany | Double-blind | 104 | 12 | TrkA inhibitor vs. Placebo |
| Kul-Panza and Berker 2010 | NR | Turkey | Double-blind | 48 | 14 | Hyaluronic acid (Orthovisc) vs. Placebo |
| Lamo-Espinosa et al 2016 | 2012-2015 | Spain | Open-label | 30 | 52 | Hyaluronic acid (Hyal-One) vs. Hyaluronic acid (Hyal-One) + Mesenchymal stem cells 10 million vs. Hyaluronic acid (Hyal-One) + Mesenchymal stem cells 100 million |
| Lee et al 2015 | 2011-2013 | Multinational: South Korea, USA | Single-blind | 54 | 24 | IA NSAIDs + Hyaluronic acid (Ketoracin+Hyal) vs. Hyaluronic acid (Hyal) |
| Lee et al 2011 | NR | South Korea | Single-blind | 43 | 16 | TissueGene-C vs. Placebo |
| Lee et al 2019 | 2015-2016 | South Korea | Double-blind | 24 | 24 | Mesenchymal stem cells (JOINTSTEM) vs. Placebo |
| Leighton et al 2014 | 2007-2008 | Multinational: Canada, Sweden, UK | Double-blind | 442 | 26 | Hyaluronic acid (Durolane) vs. Corticosteroid (Depo-Medrol) |
| Lertwanich and Lamsam 2016 | 2011 | Thailand | Open-label | 20 | 26 | Hyaluronic acid (Ostenil Plus) vs. control |
| Lin et al 2019 | 2014-2015 | Taiwan | Double-blind | 53 | 52 | Platelet-rich plasma vs. Hyaluronic acid vs. Placebo |
| Lisi et al 2018 | 2010-2014 | Italy | Open-label | 58 | 52 | Platelet-rich plasma vs. Hyaluronic acid |
| Lohmander et al 2014; Eckstein et al 2015 | 2009-2010 | Multinational: Bulgaria, Canada, Croatia, Finland, Germany, Poland, Serbia, South Africa, Sweden | Double-blind | 192 | 52 | Sprifermin single ascending dose 10 μg vs. Sprifermin single ascending dose 30 μg vs. Sprifermin single ascending dose 100 μg vs. Sprifermin multiple-ascending dose 10 μg vs. Sprifermin multiple-ascending dose 30 μg vs. Sprifermin multiple-ascending dose 100 μg vs. Placebo |
| Lomonte et al 2015 | 2010-2013 | Brazil | Double-blind | 100 | 24 | Corticosteroid (triamcinolone hexacetonide) vs. Corticosteroid (methylprednisolone) |
| Lopes de Jesus et al 2017 | 2010-2015 | Brazil | Double-blind | 98 | 16 | Ozone vs. Placebo |
| Louis et al 2018 | 2013-2016 | France | Double-blind | 54 | 26 | Platelet-rich plasma vs. Hyaluronic acid |
| Lu et al 2019 | 2013-2016 | China | Single-blind | 53 | 52 | Mesenchymal stem cells (Re-Join) vs. Hyaluronic acid (ARTZ) |
| Maheu et al 2019 | 2011-2012 | France | Double-blind | 288 | 26 | Hyaluronic acid (Structovial) vs. Hyaluronic acid (Synvisc) |
| Maheu et al 2011 | NR | Multinational: France, Belgium, Poland, Czech Republic, Estonia | Double-blind | 276 | 24 | Hyaluronic acid (Ostenil Plus) vs. Hyaluronic acid (Synvisc One) |
| Maia et al 2019 | NR | Brazil | Open-label | 44 | 26 | Hyaluronic acid (Orthovisc) vs. Hyaluronic acid (Orthovisc) + Corticosteroid vs. Corticosteroid |
| Martin et al 2016 | 2013-2015 | Italy | Double-blind | 60 | 26 | Collagen (Collagen MD-Knee) vs. Hyaluronic acid (Supartz) |
| Matas et al 2019 | 2015-2017 | Chile | Double-blind | 29 | 52 | Mesenchymal stem cells vs. Mesenchymal stem cells + Placebo vs. Hyaluronic acid (Durolane) |
| McAlindon et al 2017 | 2011-2015 | USA | Double-blind | 140 | 104 | Corticosteroid (Kenalog) vs. Placebo |
| McAlindon et al 2018 | 2014-2016 | Multinational: USA, Denmark, Czech Republic | Double-blind | 176 | 24 | Botulinum toxin type A (Botox) vs. Botulinum toxin type A (Botox) vs. Placebo |
| McGrath et al 2013 | NR | UK | NR | 182 | 52 | Hyaluronic acid (Durolane) vs. Hyaluronic acid (Synvisc) |
| Mendes et al 2019 | 2016 | Brazil | Double-blind | 105 | 12 | Botulinum toxin type A (Botox) vs. Corticosteroid vs. Placebo |
| Montanez-Heredia et al 2016 | 2014 | Spain | Double-blind | 53 | 26 | Platelet-rich plasma vs. Hyaluronic acid (Adant) |
| Navarro-Sarabia et al 2011 | 2003-2009 | Spain | Double-blind | 306 | 174 | Hyaluronic acid (Adant) vs. Placebo |
| Palmieri et al 2013 | NR | Italy | Double-blind | 62 | 26 | Hyaluronic acid (Variofill) vs. Hyaluronic acid + IA NSAIDs (Variofill + INFORCE) vs. Hyaluronic acid + Bisphosphonates (Variofill + Clasteon) |
| Parmigiani et al 2010 | NR | Brazil | Double-blind | 60 | 12 | Joint lavage + Corticosteroid vs. Placebo (air) + Corticosteroid |
| Patel et al 2013 | NR | India | Double-blind | 78 | 26 | Platelet-rich plasma (unique dose) vs. Platelet-rich plasma (2 doses) vs. Placebo |
| Paterson et al 2016 | 2011 | Australia | Double-blind | 37 | 12 | Platelet-rich plasma vs. Hyaluronic acid (Synvisc) |
| Pavelka and Uebelhart 2011 | 2007-2009 | Multinational: Czech Republic, France, Italy, Switzerland, Slovak Republic, Germany | Double-blind | 381 | 26 | Hyaluronic acid (Sinovial) vs. Hyaluronic acid (Synvisc) |
| Petrella et al 2015 | 2010-2011 | Multinational: Canada, Curacao, Belgium, The Netherlands | Double-blind | 98 | 26 | Hyaluronic acid (Hydros) vs. Hyaluronic acid + Corticosteroid (Hydros-TA) vs. Hyaluronic acid (Synvisc-One) |
| Petterson and Plancher 2019 | 2008-2009 | USA | Double-blind | 369 | 26 | Hyaluronic acid (Monovisc) vs. Placebo |
| Rahimzadeh et al 2018 | 2014-2015 | Iran | Double-blind | 42 | 26 | Platelet-rich plasma vs. Dextrose |
| Ricci et al 2017 | 2015 | Italy | Open-label | 60 | 13 | Hyaluronic acid vs. Hyaluronic acid oral therapy + Non-injectable therapy (herbal: Boswellia) (Syalox 300 Plus; Syalox 500 Plus) |
| Rossini et al 2015 | 2009 | Italy | Double-blind | 80 | 5 | Bisphosphonate vs. Placebo |
| Saccomanno et al 2016 | 2009-2013 | Italy | Open-label | 165 | 26 | Hyaluronic acid (Orthovisc) vs. Non-injectable therapy (exercise) + IA NSAIDs vs. Hyaluronic acid (Orthovisc) + Non-injectable therapy (exercise) |
| Sanchez et al 2012 | 2008-2010 | Spain | Double-blind | 176 | 24 | Plasma rich in growth factors vs. Hyaluronic acid (Synovial) |
| Sari et al 2018 | NR | Turkey | NR | 73 | 13 | Non-injectable therapy (RF neurotomy) (NeuroTherm) vs. Anesthetic + Opioid analgesic + Corticosteroid |
| Schwappach et al 2017 | 2014-2015 | USA | Double-blind | 40 | 52 | LMWF-5A vs. Placebo |
| Shapiro et al 2017; Shapiro et al 2019 | 2013-2016 [primary paper] | USA | Single-blind | 25 | 52 | Bone marrow aspirate concentrate vs. Placebo |
| Shimizu et al 2010 | 2006-2007 | Japan | Open-label | 51 | 26 | Hyaluronic acid (Artzdispo) vs. Corticosteroid (Decadron) |
| Shrestha et al 2018 | 2015-2017 | Nepal | Double-blind | 117 | 12 | Corticosteroid vs. Placebo |
| Simental-Mendia et al 2019 | 2015-2016 | Mexico | Double-blind | 35 | 48 | Platelet-rich plasma vs. Non-injectable therapy (acetaminophen) |
| Simental-Mendia et al 2016 | 2013-2015 | Mexico | Open-label | 75 | 24 | Platelet-rich plasma (Triple) vs. Platelet-rich plasma (Single) |
| Smith 2016 | NR | USA | Double-blind | 30 | 52 | Leukocyte poor-platelet-rich plasma vs. Placebo |
| Song et al 2018 | 2013-2015 | Multinational: China, Australia | Double-blind | 18 | 12 | Mesenchymal stem cells (low dose 1 × 10^7) vs. Mesenchymal stem cells (mid-dose 2 × 10^7) vs. Mesenchymal stem cells (high-dose 5 × 10^7) |
| Stevens et al 2019 | 2015-2017 | USA | Double-blind | 175 | 12 | Placebo vs. Trans-Capsaicin (CNTX-4975) + Anesthetic vs. Trans-Capsaicin (CNTX-4975) |
| Strand et al 2012; Strand et al 2016 | 2006-2007 [primary paper] | Multinational: USA, Japan | Double-blind | 379 | 13 | Hyaluronic acid (Gel-One) vs. Placebo |
| Sun et al 2017 | 2014-2015 | Taiwan | Double-blind | 132 | 26 | Hyaluronic acid (HYAJOINT Plus) vs. Hyaluronic acid (Synvisc One) |
| Suppan et al 2017 | NR | Malaysia | Open-label | 130 | 13 | Hyaluronic acid 5 ml (GO-ON) vs. Hyaluronic acid 2.5 ml (GO-ON) |
| Takamura et al 2019 | 2013-2016 | Multinational: USA, Japan | Double-blind | 308 | 26 | Hyaluronic acid (Gel-One) vs. Placebo |
| Tammachote et al 2016 | 2011-2013 | Thailand | Double-blind | 99 | 26 | Hyaluronic acid (Synvisc) vs. Corticosteroid + Anesthetic |
| Van der Weegen et al 2015 | NR | The Netherlands | Double-blind | 196 | 26 | Hyaluronic acid (Fermathron plus) vs. Placebo |
| Vanelli et al 2010 | 2006-2007 | Italy | Double-blind | 59 | 13 | Polynucleotides vs. Hyaluronic acid (Synovial) |
| Vaquerizo et al 2013 | 2011 | Spain | Open-label | 96 | 48 | Plasma rich in growth factors vs. Hyaluronic acid (Durolane) |
| Vega et al 2015 | 2012-2014 | Spain | Double-blind | 30 | 52 | Mesenchymal stem cells vs. Hyaluronic acid (Durolane) |
| Wang 2018 | 2015-2016 | China | Open-label | 56 | 4 | TNF inhibitor vs. Hyaluronic acid |
| Wu et al 2018 | 2014-2016 | Taiwan | Double-blind | 20 | 26 | Platelet-rich plasma vs. Placebo |
| Xin et al 2016 | 2009 | China | Double-blind | 229 | 6 | Hyaluronic acid (Adant) vs. Hyaluronic acid (ARTZ) |
| Yang et al 2015 | 2012-2013 | China | Open-label | 40 | 6 | Hyaluronic acid vs. Placebo |
| Yavuz et al 2012 | NR | Turkey | Open-label | 120 | 12 | Placebo vs. Corticosteroid (methyl-prednisolone acetate) vs. Corticosteroid (betamethasone) vs. Corticosteroid (triamcinolone acetonide) |
| Yazici et al 2017 | 2014-2015 | USA | Double-blind | 61 | 24 | Wnt inhibitor 0.03mg/2mL vs. Wnt inhibitor 0.07mg/2mL vs. Wnt inhibitor 0.23mg/2mL vs. Placebo |
| Yilmaz 2019 | 2016 | Turkey | Open-label | 90 | 26 | IA NSAIDs vs. Corticosteroid vs. Corticosteroid + IA NSAIDs |
| Zhang et al 2015 | 2011-2012 | China | Double-blind | 349 | 26 | Hyaluronic acid (ARTZ) vs. Hyaluronic acid (Durolane) |
| Zhao et al 2019 | 2015-2018 | China | Double-blind | 18 | 48 | Mesenchymal stem cells (low dose 1 × 10^7) vs. Mesenchymal stem cells (mid-dose 2 × 10^7) vs. Mesenchymal stem cells (high-dose 5 × 10^7) |
